# Mixed Emotions and Coping: The Benefits of Secondary Emotions

**DOI:** 10.1371/journal.pone.0103940

**Published:** 2014-08-01

**Authors:** Anna Braniecka, Ewa Trzebińska, Aneta Dowgiert, Agata Wytykowska

**Affiliations:** Department of Psychology, University of Social Sciences and Humanities, Warsaw, Poland; University of Granada, Spain

## Abstract

The existing empirical literature suggests that during difficult situations, the concurrent experience of positive and negative affects may be ideal for ensuring successful adaptation and well-being. However, different patterns of mixed emotions may have different adaptive consequences. The present research tested the proposition that experiencing a pattern of secondary mixed emotion (i.e., secondary emotion that embrace both positive and negative affects) more greatly promotes adaptive coping than experiencing two other patterns of mixed emotional experiences: simultaneous (i.e., two emotions of opposing affects taking place at the same time) and sequential (i.e., two emotions of opposing affects switching back and forth). Support for this hypothesis was obtained from two experiments (Studies 1 and 2) and a longitudinal survey (Study 3). The results revealed that secondary mixed emotions predominate over sequential and simultaneous mixed emotional experiences in promoting adaptive coping through fostering the motivational and informative functions of emotions; this is done by providing solution-oriented actions rather than avoidance, faster decisions regarding coping strategies (Study 1), easier access to self-knowledge, and better narrative organization (Study 2). Furthermore, individuals characterized as being prone to feeling secondary mixed emotions were more resilient to stress caused by transitions than those who were characterized as being prone to feeling opposing emotions separately (Study 3). Taken together, the preliminary results indicate that the pattern of secondary mixed emotion provides individuals with a higher capacity to handle adversity than the other two patterns of mixed emotional experience.

## Introduction

Based on the circumstances, it is possible to feel both positive and negative emotions at the same time [1]. Mixed emotions may arise in bittersweet situations, such as winning a disappointing prize or remembering a lost love with warmth and joy. Experiencing mixed emotions seems to be a more relevant affective response to such affectively complex events than a one-valence emotion because it more accurately represents the concurrent positive and negative aspects of the event. Mixed emotional experience seems to be particularly beneficial in stressful situations because in such a circumstances, it is impossible to avoid the negative affect associated with aversive events, while a bit of positive affect may help to ameliorate the negativity experienced. The adaptive function of mixed emotions may manifest in a lowering of the negativity embodied in an aversive event: the positive affective context changes the experience of the negative emotion by reducing its physiological arousal, without eliminating the experience of the negative emotion itself [2]. Thus, experiencing mixed emotions seems to allow a decrease in distress, but it does not interfere with the emotions’ informative function. Moreover, Larsen and colleagues [3] propose that “taking the good with the bad” might actually benefit individuals during difficult times by allowing them to confront adversity and ultimately find meaning in life’s stressors, as well as feeling better.

Accordingly, there is some empirical evidence indicating that experiencing positive and negative affects concurrently may be ideal in terms of coping with difficult life situations. For example, Tugade and Fredrickson [4] demonstrated that the positive emotional states added to negative feelings experienced in the face of aversive events play a crucial role in enhancing coping resources. In addition, there is evidence to support the idea that expressing both positive and negative affects in the face of bereavement reduces grief over time [5] and that adding positive feelings to an intensely negative psychological state when bereaved promotes coping efficiency [6]. Similarly, when experiencing the loss of a loved one, allowing positive memories to be experienced alongside sadness has led to a healthier bereavement process [7]. Furthermore, recent empirical evidence has indicated that mixed emotional experience may have a positive relationship with psychological well-being in the context of psychotherapy [8] by showing that experiencing a concurrent mixture of happiness and sadness over the course of treatment preceded improvements in psychological well-being.

Taken together, the empirical literature reviewed above offers evidence for the adaptive role that experiencing positive and negative emotions at the same time can play in coping with adversity. However, none of the research has systematically examined the benefits that mixed emotional experiences may have with respect to their different “internal structures.” It should be noted that mixed emotions are considered to have a complex nature [9], and their various patterns may have different adaptive consequences. For instance, Oceja and Carrera [10] detected the existence of at least four patterns of mixed emotional experiences: sequential, prevalence, inverse, and highly simultaneous. The sequential pattern occurs when one emotion appears first and is then replaced by a second emotion of opposing valence. The prevalence pattern is present when two opposing emotions align on a concurrent course, but one is of high intensity and the other is of low intensity. The inverse pattern takes place when positive and negative emotions evolve in an inverse fashion, i.e., when the intensity of one emotion gradually increases, the intensity of the other decreases. Finally, the highly simultaneous pattern occurs when both opposing emotions move in a simultaneous way, usually throughout the entire emotional episode. It is worth noting that research has shown that mixed emotions are associated with certain levels of affective ambivalence and tension, which varies according to the pattern experienced [10].

It seems that despite the previously mentioned benefits of mixed emotional experiences, the ambivalence associated with feeling two opposing affects may supersede their advantages. Thus, in order to gain the maximum benefits from experiencing positive and negative emotions at the same time, it seems necessary to reduce the ambivalence resulting from their co-occurrence. This study was undertaken with the supposition that this happens in some cases in which secondary emotions combine opposing affects. Secondary emotions are discrete and more complex than primary ones. They include cognitive appraisals and refer to a person’s mental model of himself or herself and others [11]. Some of them may embrace both positive and negative affects concurrently, and such secondary emotions are quite numerous [12]; among the most frequently referred to are nostalgia [13], empathy [14], [15], poignancy [16], awe [17], and tenderness [18]. It is possible that in these cases of secondary emotions, both opposing affects co-occur so succinctly that they blend into a feeling with a distinctive quality and might be considered as a specific type of mixed emotions – secondary mixed emotions. Thus, we propose that secondary mixed emotions may be another pattern of mixed emotional experience, and in the present studies, we focus on the various consequences of secondary mixed emotions for coping, as well as those of two other patterns of mixed emotional experience: sequential and simultaneous.

The sequential pattern takes place when two emotions of opposing affects switch in such a way that one single-valenced emotion appears first and is then replaced by a second single-valenced emotion [9]. For example, the individual may first feel a negative emotion (e.g., sadness) and subsequently feel a positive emotion (e.g., love or joy). The simultaneous pattern also involves feeling two opposing affects in the form of two separate emotions, but it differs in terms of the internal structure of the entire emotional experience because in this case, the positive and negative emotions move in a simultaneous way [9]. For instance, the individual may feel a positive emotion (e.g., love or joy) that runs in a concurrent course with a negative emotion (e.g., sadness). It is worth noting that both the sequential and simultaneous patterns involve feeling two opposing affects in a separate way, that is, in the form of two discrete single-valenced emotions that are experienced as at a high or moderate intensity [10]. Thus, the entire mixed emotional episode produces high levels of ambivalence and subsequent tension. The pattern of secondary mixed emotion combines positive and negative affects within one emotion and thereby involves an integrated way of feeling opposing affects. In other words, in this case, instead of feeling different emotions separately, the individual is able to feel one affective state in which opposing affects are blended into a single emotion. For example, instead of feeling positive (e.g., love) and negative (e.g., sadness) emotions separately, the individual might feel one secondary mixed emotion (e.g., lost love in the form of a nostalgic experience). Thus, the pattern of secondary mixed emotions has a distinctive quality. It assimilates opposing affects with meaningful integrity, which may subsequently reduce ambivalence and tension experienced.

It may be hypothesized that experiencing positive and negative affects together in the form of a secondary mixed emotion, rather than experiencing these affects separately in the form of two single-valenced emotions, as in the case of other patterns of mixed emotions, is more advantageous in the process of coping. As already mentioned, experiencing opposing affects separately is related to high levels of emotional ambivalence and tension, which might result in the impairment of the motivational and informative functions of emotions and thus reduce the ability to cope properly during stressful situations. In contrast, experiencing opposing affects within one discrete emotion – a secondary mixed emotion – provides a way of integrating their co-occurrence and may thus reduce ambiguity, which could provide proper orientation during a stressful situation, provoke adequate behavioral tendencies, and motivate appropriate action. In other words, in the case of a secondary mixed emotion, the ambivalence and tension resulting from opposing affects seems to be less disruptive and consequently to enable more efficient coping than the sequential and simultaneous patterns.

Additionally, there is strong empirical support suggesting that generating a linguistic label for a current emotional experience, which is more feasible for secondary mixed emotions than for other patterns of mixed emotions, tends to dampen the emotional impact of that experience [19], [20], [21]. Thus, the ability to find a single word that describes a specific emotion, such as nostalgia, poignancy, awe or tenderness, and thus verbalize one’s ambivalent experience may help to regulate that experience and reduce tension. Moreover, secondary mixed emotions, when compared with other patterns of mixed emotions, seem to represent a form of person-oriented emotional regulation [22]. The important signatures of this type of emotional regulation are holistic focus, contextual sensitivity, and integration manifested in the coordinated functioning of systems that are regarded as antagonistic, such as positive and negative affects. Person-oriented emotional regulation maintains the integrity of the overall personality system, increases the congruence between implicit and explicit self-aspects, and is believed to down-regulate emotional distress, which improves both physical and psychological functioning. With respect to the various analogies between person-oriented emotional regulation and experiencing secondary mixed emotions, similarly desirable effects seem to occur when an individual experiences this pattern in stressful life situations.

Considering all this, we hypothesized that secondary mixed emotions are more beneficial in terms of coping than other patterns of mixed emotions that involve feeling the opposing affects separately. The present three studies test this hypothesis by comparing secondary mixed emotions with two other patterns of mixed emotions (sequential and simultaneous) with respect to functionality. Two experiments address the motivational and informative impacts of mixed emotional experiences. The third study extends this research from the laboratory into real life and focuses on the individual disposition to feel secondary mixed emotions. We examined whether individuals prone to feeling secondary mixed emotions, instead of feeling opposing emotions separately, were more resilient to the stress resulting from important life transitions.

In this study, we decided to use nostalgic experience as an example of the pattern of secondary mixed emotions because nostalgia is considered to be the most understood secondary emotion that consists of both positive and negative affects. Nostalgic experience is defined as a sentimental longing for the past that is a fusion of negative emotions (typically sadness) and positive emotions (typically love or joy) [23]. Therefore, it can be assumed that in the face of the adversity, such as parting from a loved one, an individual can feel love and sadness as separate emotions occurring sequentially or simultaneously, or he or she can experience them as being blended into a single secondary mixed emotion – nostalgia for past love. Accordingly, it is hypothesized that nostalgic experience, as compared with other patterns of mixed sadness and love, favors problem solving, quick decision making, and solution-oriented action.

## Study 1

In Study 1, we focused on the motivational function of emotions, which is considered to play a particularly important role in coping [24]. The goal was to examine the impact of various patterns of mixed emotions on coping readiness. Experiencing both positive and negative affects at the same time seems to improve coping processes because positive affects that co-occur in stressful situations associated with negative feelings have been shown to foster a broader perspective on problems, sight beyond the immediate stressors, and the generation of multiple courses of action [25]. All of these effects enhance creativity [26] and facilitate rebounding from stressful emotional experiences [4]. However, as mentioned previously, the co-occurrence of positive and negative affects in the form of separate opposing emotions produces high levels of ambivalence and tension and may thus lead to cognitive and behavioral impairment.

The action-readiness triggered by emotions in stressful situations mobilizes energy and gives direction to behavior, thus promoting quick and decisive coping [27], [28]. Therefore, it is likely that in this regard, the integration of opposing affects into a feeling of distinctive quality promotes optimal functional utility by reducing ambivalence and tension. Accordingly, we hypothesized that secondary mixed emotions, as compared to two other patterns of mixed emotions, simultaneous and sequential, enhance problem solving by encouraging a quick response and solution-oriented actions.

### Method

#### Ethics Statement

“I authorize the implementation of a research project entitled ‘Co-occurrence of positive and negative affect and functionality of emotions’ conducted by Anna Chojnacka-Braniecka which received a positive opinion of The USSH Ethics Committee on Ethics of Empirical Research Involving People as Research Subjects. A copy of the application no 10/II/07-08 submitted to the Commission on 31.01.2008 constitutes an integral part of this statement.” The full name of the ethics committee: “The University of Social Sciences and Humanities Ethics Committee on Ethics of Empirical Research Involving People as Research Subjects”. The committee acts on the basis of the Resolution of the Senate of the University of Social Sciences and Humanities passed on 20th June 2006 as amended. The USSH Ethics Committee on Ethics of Empirical Research Involving People as Research Subjects specifically approved this study. The participants provided their written informed consent to participate in the study.

#### Participants and procedure

One hundred and eleven undergraduates (63 men) with an age range of 20 to 26 years (*M* = 22.32, *SD* = 2.21) participated in the experiment. Participants received course credit.

Using computer, the experiment was conducted in groups of 15 members each. The experimenter introduced the study as an “investigation of individual differences in imagination and decision making.” Participants’ comments during debriefing indicated that this framing was successful. Participants were randomly assigned to three experimental conditions: sequentially mixed emotions (love and sadness co-occurring sequentially), simultaneously mixed emotions (love and sadness co-occurring simultaneously), and secondary mixed emotion (love and sadness blended into nostalgic experience). After the participants gave informed consent, they were asked to read a text aimed at inducing one of the three patterns of mixed love and sadness. Next, they were presented with a choice task designed to assess the motivational aspect of coping efficiency: engaging in problem-solving actions and making faster decisions regarding coping strategy. Finally, the participants completed a short manipulation check and reported intensity of experienced emotions. The experimenter then debriefed the participants. The materials from each study and the data can be obtained by contacting the corresponding author.

#### Pattern of mixed emotions manipulation

There is great variation among the types of elicitations used to manipulate emotional states. In this study, we used reading a story to induce one of the three patterns of mixed emotions. This choice was made in order to control the affective content of the emotional reactions. Thus, we wanted to avoid methods that relied on real events or recalled experiences because of the difficulties involved in structuring material based on real-life experiences and controlling how these stimuli influence the affective content of emotional reactions. According to a recent meta-analysis of emotion induction techniques [29], reading a text that includes imagined scenarios involving the experiences of a protagonist may be an optimal elicitor when it is important to standardize the presentation and content of the material. Therefore, we decided that in our study, this technique would be an especially convenient form of elicitation because it enables the accurate manipulation of the patterns of mixed emotions across experimental conditions.

Participants were asked to read a short romantic story and to empathize with the protagonist. They were asked to get into the feeling of the story and to try to imagine what they would feel if they were in the situation described. The text of the story consisted of two parts. The first part included a description of the protagonist’s situation, and the second part included his/her emotional response to it. The aim of the first part, which was the same in all experimental conditions, was to introduce the participants to the context of the protagonist feelings. Therefore, it was rather short and written in the third person singular. After a long heartbreaking separation from a beloved boyfriend/girlfriend (depending on the sex of the participant) who was working in a remote country, the protagonist receives a message stating that the boyfriend/girlfriend did not obtain an expected job in their town as expected and would be forced to stay abroad for good. The purpose of the second part of the story was to induce mixed emotional experiences. Participants were presented with one of three descriptions of the protagonist’s emotional response to this situation (written in the first person singular) with each representing one of the three patterns of mixed emotions: love and sadness sequentially (sequentially mixed emotions; Seqentially ME), love and sadness simultaneously (simultaneously mixed emotions; Simultaneously ME), and love and sadness blended into a nostalgic experience (secondary mixed emotion; Secondary ME). Before reading the descriptions of emotional responses, the participants were instructed to put themselves into the situation and feel the experiences described. They were asked to read each sentence slowly and carefully and to try to empathize with the protagonist’s feelings as intensely as possible.

In order to induce similar emotional states in all experimental conditions, with the exception of the way in which opposing affects were combined, we have made efforts to elicit affectively balanced experiences of similar content. Therefore, all three descriptions of emotional responses were counterbalanced in regard to positive and negative expressions. In other words, the description consisted of eleven positively and eleven negatively valenced sentences in each condition, and both affectively opposing parts of the text were of a similar length (89 words in the positively valenced part and 87 words in the negatively valenced part in each condition). In addition, each description included similar content motifs, such as disappointment in not being offered a job, a wish to bring back past time, remembering moments spent with loved ones, walking with someone, listening to music, and a vacation by the sea. Nostalgic experience was elicited by sorrowfully recalling happy memories of the time spent with the boyfriend/girlfriend. In line with previous research on the content of nostalgia [23], our story depicted a sense of redemption in the definitive parting from the boyfriend/girlfriend by inducing a subsequent triumph: recalling the strong emotional bond with him/her through loving memories of being together. To elicit the two other patterns of mixed emotions, the same story was composed in line with a design tested by Carrera and Oceja [9]. For the sequentially mixed condition, the story first presented a negative element (sorrow over the loss) and then a positive one (reminiscence of love). Because the order of emotions can play an important role in sequentially mixed experiences, the order in which love and sadness were elicited was counterbalanced. In the simultaneously mixed condition, the story switched between both elements alternately: Sentences expressing sorrow and love were combined into a description of feeling of love and sadness in parallel and with a high level of intensity. Because there are verbal and behavioral difficulties in expressing opposing emotions simultaneously [30], the protagonist thus expressed love and sadness in turns. In this condition, the same sentences as those used in the sequentially mixed condition were provided, but they were combined into a single description of feeling love and sadness together. Descriptions from all three conditions are provided in Supplementary Material S1.

#### Dependent measures

Two indicators of coping were used: (a) the choice between solution-oriented action and avoidance and (b) the latency of that choice. After reading the story, the participants were asked to choose one of two ways of reacting to the pain of being separated from a beloved person: solving the problem by making an effort to be together again (solution-oriented action) or leaving the problem behind and doing something pleasant (avoidance). Both responses were social. The solution-oriented strategy involved making contact with a friend who might help organize an inexpensive plane trip to meet the boyfriend/girlfriend, and avoidance involved going shopping with a friend. Because the participants were previously led to feel positive about the hypothetical partner and their relationship and trying to maintain and take care of close positive relationships is generally adaptive, making efforts to be together represents a more beneficial coping than avoidance. This is especially true since having satisfactory and close relationships are regarded as substantial factors in determining well-being. The time taken to choose a coping activity was recorded. Because making choices in a short period of time is an indicator of a high processing speed, which is considered to promote the quality and the accuracy of cognition as well as necessary for efficient coping, we acknowledge that shorter latencies are more adaptive than longer ones. In addition, the specificity of the choice did not require spending time in deep thought because the decision was potentially reversible (it involved an initial step toward one of two possible directions) and hypothetical (it did not relate to a participant’s real life situation).

### Results and Discussion

Before conducting inferential analyses, the reaction times were log-transformed. Then, the data from five persons were excluded from the analyses due to extremely short or long latencies (+/−3 SD). Thus, the final analyses were performed on 106 undergraduates (60 men).

#### Manipulation check

To examine the effect of mixed emotion elicitation, participants were asked to report their feelings while reading the story by choosing among four options: (a) *sentimental longing for the past*, (b) *love and sadness separately*, (c) *love and sadness simultaneously*, and (d) *none of these*. Additionally, the participants estimated the intensity of the feeling experienced while reading the descriptions of emotional responses. They were asked to indicate how intense the emotions were that they were feeling using a 0–100 scale with 0 being *not at all intense* and 100 being *as intense as I ever felt before* by marking the appropriate number on the line. A chi-square test showed a significant relationship between the target emotions and the emotions reported by the participants (χ^2^ [6; *N = *106] = 86.63, *p*<.001). Specifically, in the Secondary ME condition, 25 participants (67.6%) reported *sentimental longing for the past*, five participants (13.5%) reported *love and sadness simultaneously*, five (13.5%) reported *none of these*, and two (5.4%) reported *love and sadness sequentially*. In the Sequentially ME condition, 22 participants (61.1%) reported having felt *love and sadness sequentially*, ten participants (30.6%) reported *love and sadness simultaneously*, two participants (5.6%) reported *sentimental longing for the past*, and one (2.8%) reported *none of these*. In the Simultaneously ME condition, 29 participants (87.9%) reported having experienced *love and sadness simultaneously*, two participants (6.1%) reported *love and sadness sequentially*, one (3.0%) reported *none of these*, and one (3.0%) reported *sentimental longing for the past*. Thus, it can be assumed that in all of the conditions, most of the participants reported having felt emotions consistent with our intentions, which suggests that the experimental manipulations were effective. A one-way analysis of variance (ANOVA) demonstrated that the differences among groups in terms of the intensity of the elicited emotions were not significant (*F* [2, 103] = 1.61, *ns* [*M = *70.41, *SD = *15.51 in the Secondary ME condition; *M = *66.42, *SD = *16.12 in the Sequentially ME condition; and *M = *63.15, *SD = *19.22 in the Simultaneously ME condition).

#### Choice of coping

A chi-square test showed a significant relationship between the pattern of mixed emotions and the chosen coping activity (*χ^2^* [2; *N = *76] = 8.77, *p*<.05). For the Secondary ME condition, solution-oriented action was chosen by 29 participants (78.4%), 25 participants (69.4%) chose it for the Sequentially ME condition, and 15 participants (45.5%) chose it for the Simultaneously ME condition. Accordingly, avoidance was chosen by 18 participants (54.5%) in the Simultaneously ME condition, 11 participants (30.6%) in the Sequentially ME condition, and eight participants (21.6%) in the Secondary ME condition. Thus, according to our expectations, in the Secondary ME condition (in comparison with both the Simultaneously ME and Sequentially ME conditions), solution-oriented action was chosen more frequently. This confirms that the integration of love and sadness into one discrete emotion promotes undertaking solution-oriented actions. In addition, in the Simultaneously ME condition, avoidance was more frequently chosen than in the Secondary ME and Sequentially ME conditions and more frequently chosen than solution-oriented action, which was not observed in the other conditions.

#### Latency of making a choice

A one-way ANOVA showed the effects of the patterns of mixed emotions (*F* [2, 103] = 7.54, *p<*.01, *η* ^2^ = .13). The latency of making a choice (in milliseconds) was longer in the Simultaneously ME condition (*M = *10.05, *SD = *0.36) than in both the Secondary ME (*M = *9.70, *SD = *0.56, *p*<.05) and Sequentially ME (*M = *9.61, *SD = *0.54, *p*<.01) conditions. There was no significant difference between the Secondary ME and Sequentially ME conditions. Multiple comparisons were performed using Tukey’s HSD test. Thus, this result suggests that both secondary mixed emotions and sequentially mixed opposing emotions have a higher functionality with respect to triggering a quick response than simultaneous mixed opposing emotions. It appears that the sequential pattern of mixed emotions, because of its high level of clarity, provides good behavioral guidance and thus may support quick decision making. The beneficial impact of a secondary mixed emotion pattern in terms of making a quick choice may result from its coherent structure, which facilitates the efficient processing of information.

The results of Study 1 indicated that the pattern of secondary mixed emotion represents a more adaptive way of experiencing positive and negative emotions at the same time than both the sequential and simultaneous patterns. Specifically, the results supported our expectations that love and sadness blended into one secondary emotion would enhance problem-solving coping, which is defined as quickly choosing solution-oriented action. Furthermore, these outcomes demonstrated that the simultaneous occurrence of love and sadness might impair the motivational aspect of coping during task orientation and the accessibility of a resolution. It is likely that as demonstrated in previous research [10], the simultaneous pattern of mixed emotional experiences produces the highest level of ambivalence associated with uncomfortable tension, which is a source of vague and unstable motivational guidance.

## Study 2

In line with the assertion that the important function of emotions in the context of coping is to facilitate understanding the situation [31], [32], we designed Study 2 to verify the functionality of mixed emotions with respect to stimulating thought. Experiencing positive and negative affect at the same time seems to be particularly beneficial to cognition during the process of coping with adversity. There is plenty of evidence indicating that negative mood states impair information processing during adverse circumstances. For example, experiencing negative affect fosters a mood-congruent cognitive bias toward negative information and impairs attentional disengagement from it [33]. In addition, people influenced by negativity related to personal losses tend to attach more weight to false negatives, resulting in overly narrow categorization [34]. Thus, it is possible that experiencing positive affect would reduce the detrimental effects of negativity and thus promote a correct understanding of the situation. Moreover, the explanation of the beneficial effects of mixed emotional states on coping offered by Larsen and colleagues [3] proposes that experiencing opposing affects together allows individuals to confront adversity and subsequently find meaning in difficult life circumstances. There are evidence that positive affect joined to negative feelings allows individuals to feel better and, more importantly, broadens cognitive processing, fosters access to existing knowledge, creates openness to new information [35], and promotes the processing of emotionally ambiguous information [36]. In addition, positive affect facilitates access to positive information about the self [37] that is usually ignored [38], while positive self-regard serves as a validation of emotions as information and augments reliance on emotional cues, both positive and negative, which has been proven to have a relatively strong and long-lasting effect [39]. Thus, positive affect added to negative emotional states seems to extend its informative function via soothing the experience of stress and expanding cognitive processing.

The only problem limiting the informative function of mixed emotions in stressful situations may be the disruptive effects of tension produced by contradictory affective arousal, which can ultimately diminish cognitive effectiveness during the process of coping. Therefore, a secondary mixed emotion with low levels of ambivalence and tension may be superior to separately co-occurring opposite affects due to enhancing thinking. Accordingly, we hypothesized that people feeling a secondary mixed emotion (such as nostalgic experience) are more informed by their emotions than people experiencing other patterns of mixed emotions (love and sadness felt in the sequential or simultaneous way), that is, they construct a more elaborate narrative and have better access to self-relevant information.

### Method

#### Ethics Statement

“I authorize the implementation of a research project entitled ‘Co-occurrence of positive and negative affect and functionality of emotions’ conducted by Anna Chojnacka-Braniecka which received a positive opinion of The USSH Ethics Committee on Ethics of Empirical Research Involving People as Research Subjects. A copy of the application no 10/II/07-08 submitted to the Commission on 31.01.2008 constitutes an integral part of this statement.” The full name of the ethics committee: “The University of Social Sciences and Humanities Ethics Committee on Ethics of Empirical Research Involving People as Research Subjects”. The committee acts on the basis of the Resolution of the Senate of the University of Social Sciences and Humanities passed on 20th June 2006 as amended. The USSH Ethics Committee on Ethics of Empirical Research Involving People as Research Subjects specifically approved this study. The participants provided their written informed consent to participate in the study.

#### Participants and design

Ninety-six undergraduates (56 women) with an age range of 20 to 25 years (*M* = 22.23, *SD* = 1.72) participated in the experiment. Participants received course credit for their participation.

The experimenter introduced the study as an “investigation of individual differences in imagination and writing stories.” The overall experimental design was similar to that used in Study 1. By means of the same manipulation, participants were randomly assigned to three experimental conditions: secondary mixed emotion (Secondary ME), simultaneously mixed emotions (Simultaneously ME), and sequentially mixed emotions (Sequentially ME). Next, they were asked to write down a narrative describing the story they had just read in their own words and then fill out a self-evaluation questionnaire. Finally, the participants completed manipulation check and reported intensity of experienced emotions. The experimenter then debriefed the participants.

#### Dependent measures

Participants were presented with a computer and asked to create a narrative describing the story they had just read. The narratives were analyzed according to the number of main elements from the story plot used by the participants. It has been claimed that the more elements from the plot could be identified, the higher the elaboration and complexity of the narrative [40]. Three trained coders who were unaware of the hypothesis independently rated the participants’ narratives by counting the following elements: (a) the presence of the protagonist, (b) the presence of the protagonist’s intentions and plans, (c) the presence of the protagonist’s efforts to carry out these plans, (d) the presence of the difficulties in this process, (e) the presence of other characters, and (f) the presence of these characters’ intentions and plans. The ratings ranged from 0 (*no elements of the plot occur*) to 6 (*all elements of the plot occur*). The inter-judge reliability of the three coders was significant (Cohen’s kappa = .74, *p*<.01). Inconsistencies between the coders were resolved via discussion.

The accessibility of self-knowledge was measured based on the principles of the information-processing approach [41], suggesting that shorter processing times for self-relevant information promotes a better understanding of oneself. Thus, in the present study, the time needed to complete a computerized version of the Rosenberg Self-Esteem Scale (SES) [42] served as an index for the accessibility of self-knowledge. The SES is a self-reported ten-item questionnaire that is answered using a 4-point scale (0 to 3). A higher total score indicates a higher level of self-esteem. In this study, we were not interested in the level of self-esteem. Nonetheless, it was measured, and the obtained results are presented below the main results.

### Results and Discussion

Before conducting inferential analyses the reaction times were log-transformed. Of the 96 participants, 94 students (54 women) remained for the analyses because data from two persons were excluded due to extremely short or long latencies (+/−3 SD).

#### Manipulation check

To examine the effect of mixed emotion elicitation, participants responded to the same question that was used in Study 1. A chi-square test demonstrated that the relationship between the target emotional states and the emotional states reported by the participants was significant (*χ^2^* [6; *N = *94] = 49.86, *p*<.001). Specifically, in the Secondary ME condition, 21 participants (65.6%) reported having felt *sentimental longing for the past*, six participants (18.8%) reported *love and sadness simultaneously*, three (9.4%) reported *none of these*, and two (6.3%) reported *love and sadness sequentially*. In the Sequentially ME condition, 18 participants (62.1%) reported having experienced *love and sadness sequentially*, nine participants (31%) reported *love and sadness simultaneously*, one participant (3.4%) reported *none of these*, and one (3.4%) reported *sentimental longing for the past*. In the Simultaneously ME condition, 20 participants (60.6%) reported having experienced *love and sadness simultaneously*, seven (21.2%) reported *sentimental longing for the past*, three participants (9.1%) reported *love and sadness sequentially*, and three (9.1%) reported *none of these*. As in Study 1, the intensities of the elicited mixed emotions were at similar levels under all of the experimental conditions (*M = *65.72, *SD = *16.44 in the Secondary ME condition; *M = *57.55, *SD = *17.23 in the Sequentially ME condition; and *M = *64.61, *SD = *14.16 in the Simultaneously ME condition; the differences among the groups were not significant: *F* [2, 91] = 2.32, *ns*).

#### Level of narrative elaboration

A one-way ANOVA showed the effects of the patterns of mixed emotions (*F* [2, 91] = 18.99, *p<*.001, *η* ^2^ = .29). In line with our expectations, the narrative elaboration was higher in the Secondary ME condition (*M = *3.59, *SD = *1.41) than in both the Sequentially ME (*M = *2.17, *SD = *1.23) and Simultaneously ME (*M = *1.73, *SD = *1.15) conditions. Significant differences were found between the Secondary ME and Simultaneously ME conditions (*p*<.001) and between the Secondary ME and Sequentially ME conditions (*p*<.001). Multiple comparisons were performed using Tukey’s HSD test. Thus, experiencing positive and negative affects blended into a secondary mixed emotion, when compared with the other patterns of mixed opposing affects, promoted the highest level of narrative elaboration, which may reflect a particularly insightful understanding of social reality.

#### Latency of self-evaluation

The one-way ANOVA showed the effects of the patterns of mixed emotions (*F* [2, 91] = 17.75, *p<*.001, *η* ^2^ = .28). The latency of self-evaluation (in milliseconds) was shorter in the Secondary ME condition (*M = *9.83, *SD = *.65) than in both the Sequentially ME (*M = *10.67, *SD = *.49) and Simultaneously ME (*M = *10.62, *SD = *.70) conditions. There were significant differences only between the Secondary ME and Simultaneously ME conditions (*p*<.001) and between the Secondary ME and Sequentially ME conditions (*p*<.001). Multiple comparisons were performed using Tukey’s HSD test. Thus, according to our expectations, opposing affects blended into nostalgia, as compared with the other patterns of mixed emotions, was the most functional pattern in terms of easy access to self-knowledge. The blending of positive and negative affects into a secondary mixed emotion might be particularly relevant to self-knowledge, which is founded in the integration of both positive and negative beliefs about the self.

Additionally, although it was not the goal of the study, the level of self-esteem was measured. The internal consistency of the SES was high (*α* = .82). A one-way ANOVA showed the effects of the patterns of mixed emotions (*F* [2, 91] = 14.98, *p*<.001, *η^2^* = 0.25). Self-esteem was higher in the Secondary ME condition (*M = *25.78, *SD = *3.34) than in both the Sequentially ME (*M = *20.90, *SD = *5.12) and Simultaneously ME (*M = *20.03, *SD = *4.97) conditions. Significant differences were found between the Secondary ME and Simultaneously ME conditions (*p*<.001) and between the Secondary ME and Sequentially ME conditions (*p*<.001). Multiple comparisons were conducted by Games-Howell’s test (unequal variances). Thus, of all of the elicited patterns of mixed emotions, secondary mixed emotions created the highest level of self-esteem, whereas the sequential and simultaneous patterns had, in this regard, less beneficial impacts. It is noteworthy that comparable results were obtained by Wildschut and colleagues [23]. In experimental settings, they demonstrated that bringing to mind a nostalgic event, which represents circumstances related to experiencing secondary mixed emotions in our study, increased self-regard. In other words, secondary mixed emotions, as compared with the other patterns of mixed emotions, were the most functional experience not only in terms of easy access to self-knowledge but also in terms of improving thoughts about the self. Nonetheless, it should be noted that although we used the scale (SES) that measures state self-esteem by asking the respondents to reflect on their current feelings, the level of self-esteem is in part related to global, relatively stable feelings of self-worth and self-acceptance and cannot be explained exclusively by a current state. Therefore, the conclusions regarding the effects of induced patterns of mixed emotions on the level of self-esteem may be limited by variations in trait self-esteem. In order to confirm the impact of different patterns of mixed emotions on self-esteem, the level of this variable should be measured at two time points: before and after experimental manipulation, for instance.

Altogether, the results supported our expectations that the pattern of secondary mixed emotion represents the most advantageous way of co-occurring opposing affects in the context of cognitive effectiveness during the process of coping with an imagined stressful situation. Thus, the results of Study 2 demonstrated that experiencing positive and negative affects blended into a secondary mixed emotion enhanced the informative function of emotional experiences and that when compared with both the sequential and simultaneous patterns of mixed emotions, secondary mixed emotion generated the highest level of narrative elaboration and the fastest self-evaluation.

## Study 3

In Studies 1 and 2, the elicited negative and positive affects blended into a secondary mixed emotion increased the motivational and informative functions of the experienced emotional states. Therefore, in Study 3, we investigated the effect of the individual disposition toward feeling this pattern of mixed emotions on general resilience in real life. Resilience refers to the processes of coping with stress and adversity, which results in returning to a previous state of normal functioning or simply not showing any negative consequences [43]. Most research indicates that resilience is the effect of being able to interact with one’s environment in a way that either promotes well-being or protects against the risk factors of psychopathology [44]. In the present study, the chosen challenge was the stress of transitioning to a university, and the indicator of resilience was the ability to adjust to this considerable life adversity and return to a previous state of healthy functioning. Starting an academic education is often regarded as a period of turmoil for young people and has been associated with an increased risk of depressive symptoms, anxiety, and other psychopathological problems [45], [46]. Freshmen are faced with the necessity of managing various changes occurring in many domains of their lives at once, such as being immersed in various learning environments, greater responsibilities, and new peer groups. Although starting a new school can be difficult, some students successfully adjust to this major life change in such a way that their distress and impairment decrease over time, whereas others do not.

These individual differences in psychological adaptation can be explained by the variety of emotional responses to life difficulties. In stressful situations, people respond to a negative mood with a wide array of emotion-regulation strategies, mostly related to reducing negative affect and inducing positive affect, and the consequences of these responses depend on the adaptive value of the chosen strategies [47]–[49]. Researchers have suggested that better adjustment is related to possessing various emotional competencies, such as the capacity to experience affective complexity, which may be an important protective factor against internalizing symptomatology in the face of life stressors [50], [51]. There is evidence that accepting affective ambiguity is an individual trait associated with a tolerance for uncertainty [52], and experiencing contradictory emotions at the same time at either a moderate or high intensity is considered to be a highly advanced state of feeling because it is accessible only later in childhood [53]. Moreover, there is plenty of evidence showing that the ability to feel mixed emotions may be considered a developmental achievement attained over the entire lifespan and may be especially beneficial to quality of life [54]–[56]. Consequently, individuals who have failed in coping with an emotional crisis that resulted in psychiatric hospitalization, showed lower ability to experience positive and negative affects within one emotional state when confronted with affectively complex stimuli, such as ambivalent jokes, than the healthy control group [57], [58]. Thus, individuals disposed to feel mixed emotions in adverse situations seem to cope more efficiently than others because they experience positive affect in combination with the negative emotional states caused by a stressful event, which may provide emotional comfort. Moreover, it helps to retain aversive or unpleasant thoughts and memories within the working memory. Thus, one assimilates these cognitions within a meaningful narrative [3] and sustains these coping processes during chronic stress [59].

Nevertheless, as stated previously, mixed emotional experiences are considered to be complex in nature, which is reflected in the existence of several patterns of mixed emotions. Accordingly, it can be assumed that individual dispositions toward feeling different patterns of mixed emotions may have different adaptive consequences. Based on the results obtained in Studies 1 and 2, we hypothesized that the ability to experience the pattern of secondary mixed emotion should promote higher resilience than the disposition towards other feeling patterns. Thus, Study 3 aims to verify whether resilience to the stress of a transition depends on the disposition toward experiencing the pattern of secondary mixed emotion rather than co-occurring distinct emotions during adverse events. Assuming that people differ in their proneness to experiencing secondary mixed emotions, we hypothesized that freshmen who are disposed toward transforming negative emotion (e.g., sadness) into a secondary mixed emotion (e.g., nostalgia) would be, after some time, better off than those who experience negative and positive emotion separately. We chose sequentially co-occurring emotions for comparison because that pattern is shown to be more frequent [10], and in Study 1, it was not that much worse than the pattern of secondary mixed emotion in terms of functionality.

### Method

#### Ethics Statement

“I authorize the implementation of a research project entitled ‘Co-occurrence of positive and negative affect and functionality of emotions’ conducted by Anna Chojnacka-Braniecka which received a positive opinion of The USSH Ethics Committee on Ethics of Empirical Research Involving People as Research Subjects. A copy of the application no 10/II/07-08 submitted to the Commission on 31.01.2008 constitutes an integral part of this statement.” The full name of the ethics committee: “The University of Social Sciences and Humanities Ethics Committee on Ethics of Empirical Research Involving People as Research Subjects”. The committee acts on the basis of the Resolution of the Senate of the University of Social Sciences and Humanities passed on 20th June 2006 as amended. The USSH Ethics Committee on Ethics of Empirical Research Involving People as Research Subjects specifically approved this study. The participants provided their written informed consent to participate in the study.

#### Participants and design

The participants included 118 psychology freshmen (90 women; mean age = 22.70 years, *SD* = 2.04 years). They received course credit for their participation. The study was conducted according to a longitudinal design; it consisted of two time points: one during the second month of the academic year (Time 1) and another six months later (Time 2). At Time 2, 91 freshmen from the group remained (69 women; mean age = 20.82 years, *SD* = 1.76 years).

#### Procedure

At each time point, the participants received a booklet containing instructions and materials. At Time 1, the booklet consisted of a measurement of proneness to experiencing secondary mixed emotions (measurement of nostalgia proneness) and four questionnaires measuring dependent variables: the levels of depressive symptoms, anxiety, positivity ratio, and somatic health. At Time 2, the measurement included only a re-assessment of the dependent variables.

#### Secondary mixed emotion proneness

To assess the pattern of mixed emotions on a dispositional level, the participants were asked to empathize with a person separated from his/her boyfriend/girlfriend and then choose the emotional response they identified as being more natural for them in such an adverse life situations. Therefore, we presented a story intended to elicit sadness at first. Then, two alternative continuations were provided that aimed to elicit mixed emotional reactions: the feeling of a secondary mixed emotion and the feeling of positive and negative emotions occurring sequentially. It has been claimed that reading a story might be an optimal method for this study because its advantages include standardized materials and presentation, which was an important requirement of our assessment [29]. The participants were instructed to put themselves into the situation and to empathize with the protagonist as intensely as possible. They were asked to try to imagine what they would feel if they were in such circumstances. The alternative emotional responses represented two patterns of mixed emotions: the pattern of secondary mixed emotion (nostalgic experience) and the sequential pattern (sadness and love or love and sadness – the parts of the text eliciting negative or positive emotions were presented in a counterbalanced order). The story was the same as that used in Study 1 and Study 2 to evoke various patterns of mixed emotions. The only change was the task. In the previous studies, we asked the participants to feel the loss and reaction in the same way as the protagonist did; however, in the presented study, the participants were asked to feel the same loss as the protagonist and then reveal their own emotional reaction to it.

#### Depressive symptoms

The Beck Depression Inventory (BDI-II) [60] was used; it is a self-report inventory that contains 21 questions relating to symptoms of depression, such as hopelessness, irritability, feelings of guilt or being punished, fatigue, weight loss, and lack of interest in sex, with each answer being scored on a scale of 0 to 3. A higher total score indicated more depressive symptoms (*α* = .91).

#### Anxiety

The part of the State-Trait Anxiety Inventory measuring a temporary “state of anxiety” (STAI-S) [61] was administered. This is a 20-item self-report measure of anxiety symptoms, such as feelings of fear and tension and autonomic nervous system hyperactivity. Individuals rate how they currently feel by using a 4-point scale (*α* = .89).

#### Positivity ratio

The participants completed the Positive and Negative Affect Schedule (PANAS) [62], which consists of two 10-item scales composed of adjectives measuring positive affect (PA; e.g., alert, active, and interested) and negative affect (NA; e.g., jittery, hostile, and ashamed). The participants indicated to what extent they currently experienced these feelings by using a 5-point scale (α = .80 and.87 for the PA and NA, respectively). The ratio of the PA to NA scores was a positivity ratio [63].

#### Somatic symptoms

The somatic subscale of the General Health Questionnaire (GHQ-28-A) [64] was used. The GHQ-28 is a self-report screening test for detecting the risk of developing psychopathological problems. The administered subscale was developed to measure physical health complaints, such as feelings of exhaustion and fatigue, having hot or cold spells, being ill, or having a headache with pressure in the head. This subscale contains seven items rated on a 4-point scale. A higher total score indicated more somatic symptoms (*α* = .76).

### Results and Discussion

#### Emotion elicitation check

The participants were asked to report their feelings while reading the story and while reading a description of alternative responses separately by choosing among five options: (a) *sadness*, (b) *sentimental longing for the past*, (c) *love followed by sadness*, (d) *sadness followed by love*, and (e) *none of these*. Sixty two (68.2%) participants reported feeling the expected emotions: *sadness* while reading the story and a *sentimental longing for the past* or *love followed by sadness* (*or sadness followed by love*) while reading the nostalgic response and the sequentially mixed love and sadness response, respectively. The remaining 29 (31.8%) participants reported having felt varied combinations of emotions that were inconsistent with our intentions. Therefore, it can be concluded that mixed emotions were effectively elicited. The results demonstrated that most of the participants showed a disposition toward feeling secondary mixed emotions rather than co-occurring distinct emotions. Fifty-seven freshmen (62.5%) declared experiencing secondary mixed emotion to be more natural to them, whereas 34 freshmen (37.5%) declared that experiencing positive and negative emotion sequentially was more natural to them.

In Time 1, 75 participants (63.6%) declared experiencing secondary mixed emotion to be more natural to them, whereas 43 participants (36.4%) declared experiencing opposing emotions sequentially to be more natural to them. In other words, similar percentage of participants dropped out from each mixed emotions pattern: 24.0% from the pattern of secondary mixed emotion and 20.9% from the sequential pattern.

We conducted separate repeated-measures ANOVAs for each dependent variable, using time (Time 1 *vs*. Time 2) as the within-subject factor and the pattern of mixed emotions (secondary mixed emotions *vs*. the sequential pattern) as a between-subject factor. All conducted simple effects analyses were Bonferroni corrected.

#### Depressive symptoms

**Figure 1:**
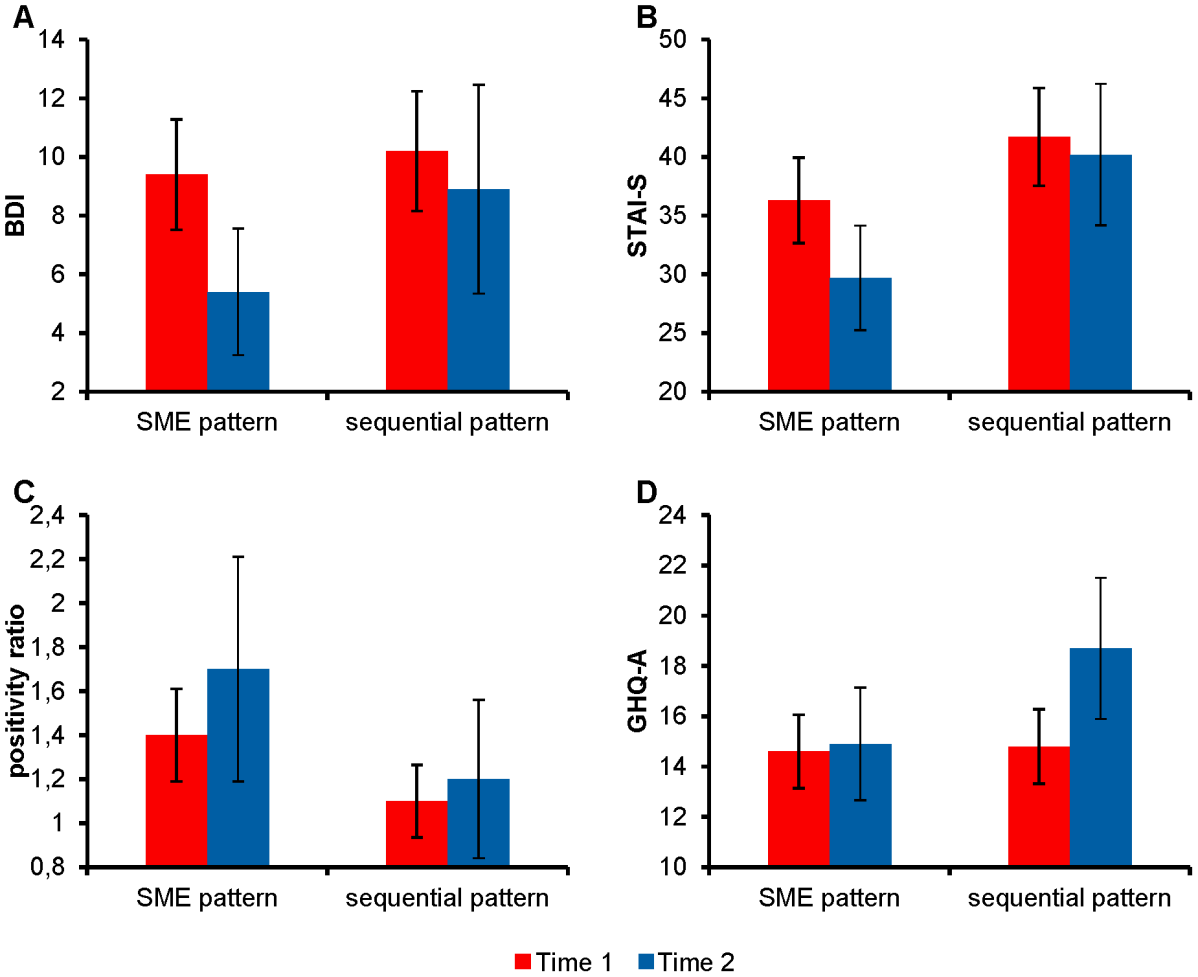
Means of resilience measures for the patterns of mixed emotions at two time points. Display of mean values of depressive symptoms (BDI) (A), anxiety symptoms (STAI-S) (B), positivity ratio (C), and somatic symptoms (GHQ-A) (D) for the proneness to experiencing the pattern of secondary mixed emotion (SME pattern) and the proneness to experience the sequential pattern of mixed emotions (sequential pattern) at two time points - in the second month of the academic year and six months later. Bars are standard deviations.

The analyses yielded a main effect for time (*F* [1, 89] = 14.64, *p*<.001, *η* ^2^ = .14). The total BDI score decreased from Time 1 (*M = *9.73, *SD = *4.63) to Time 2 (*M = *6.71, *SD = *5.61). A significant effect was also found for the pattern of mixed emotions (*F* [1, 89] = 6.68, *p*<.005, *η* ^2^ = .07). As predicted, participants who declared that they experienced secondary mixed emotion reported a lower level of depressive symptoms (*M = *7.41, *SD = *4.91) than participants who declared experiencing positive and negative emotions sequentially (*M = *9.57, *SD = *5.13). These effects were qualified, however, by the interaction between these two factors (*F* [1, 89] = 4.02, *p*<.05, *η* ^2^ = .04). A decrease in depressive symptoms between Time 1 and Time 2 was observed in the group experiencing secondary mixed emotions (*F* [1, 89] = 22.76, *p*<.01, *η* ^2^ = .24) but not in the group experiencing opposing emotions sequentially (*F* [1, 89] = 1.32, *ns*; see Figure 1).

#### Anxiety

With regard to anxiety symptoms, there was also a main effect for time (*F* [1, 89] = 15.98, *p*<.001, *η* ^2^ = .15). The anxiety score decreased from Time 1 (*M = *38.36, *SD = *7.30) to Time 2 (*M = *33.65, *SD = *9.35). The analyses also indicated a main effect for the pattern of mixed emotions (*F* [1, 89] = 6.68, *p*<.005, *η* ^2^ = .07). In participants experiencing secondary mixed emotion, anxiety was lower (*M = *33.03, *SD = *6.84) than in participants experiencing positive and negative emotion sequentially (*M = *40.99, *SD = *8.15). However, these effects were qualified by a two-way interaction (*F* [1, 89] = 6.36, *p*<.05, *η* ^2^ = .06). The participants experiencing the pattern of secondary mixed emotions demonstrated less anxiety from Time 1 to Time 2 (*F* [1,89] = 28.45, *p*<.001, *η* ^2^ = .24), whereas there was no such effect in the group with a sequential pattern (*F* [1, 89] = .86, *ns*; see Figure 1).

#### Positivity ratio

There was a main effect for time (*F* [1,89] = 9.86, *p*<.001, *η* ^2^ = .10). The positivity ratio increased from Time 1 (*M = *1.30, *SD = *0.64) to Time 2 (*M = *1.57, *SD = *0.78). There was also a main effect for the pattern of mixed emotions (*F* [1, 89] = 10.35, *p*<.01, *η* ^2^ = .14). As predicted, participants experiencing the pattern of secondary mixed emotion acquired higher positivity ratios (*M = *1.55, *SD = *0.65) than participants feeling the sequential pattern (*M = *1.17, *SD = *0.64). There was also a two-way interaction (*F* [1, 89] = 4.29, *p*<.05, *η* ^2^ = .05). In the group experiencing secondary mixed emotions, positivity increased from Time 1 to Time 2 (*F* [1, 89]) = 18.18, *p*<.001, *η* ^2^ = .17), whereas there was no such effect in the group with a sequential pattern of positive and negative emotions (*F* [1, 89] = 0.45, *ns*; see Figure 1).

#### Somatic symptoms

The GHQ-A score increased from Time 1 (*M = *14.64, *SD = *4.21) to Time 2 (*M = *16.37, *SD = *6.34; *F* [1,89] = 6.69, *p*<.01, *η* ^2^ = .07). There was also a main effect for the pattern of mixed emotions (*F* [1, 89] = 6.30, *p*<.05, *η* ^2^ = .06). The participants experiencing secondary mixed emotions reported fewer somatic symptoms (*M = *14.79, *SD = *4.62) than participants feeling positive and negative emotions sequentially (*M = *16.76, *SD = *5.96). These effects were, however, qualified by a two-way interaction (*F* [1, 89] = 4.49, *p*<.05, *η* ^2^ = .04; see Figure 1). An increase in somatic symptoms between Time 1 and Time 2 occurred in the group feeling opposing emotions sequentially (*F* [1, 89] = 8.83, *p*<.01, *η* ^2^ = .09). There were no such effects in the group experiencing secondary mixed emotions (*F* [1, 89] = .14, *ns*). These results corresponded with the empirical evidence showing that the impact of stress on one’s physical health is often delayed because of the specificity of immune system functioning [65]–[67]. Therefore, at the start of the academic year, the freshmen did not yet report an increase in physical health problems, although some of them already showed mental health impairment. Over time, the freshmen feeling opposite emotions sequentially reported more physical health complaints, whereas those feeling secondary mixed emotions did not. It should be noted, however, that the measure of somatic symptoms was based on self-reports exclusively because it is well-known that the perception of these symptoms may result from the existence of objective problems with physical health as well as from an individual’s tendency to generate somatic complaints. Thus, there is a possibility that freshmen who feel sequentially mixed emotions are simply more sensitive to any change in their somatic status, so they may just be more prone to list physical health complaints. Although we are not aware of any research suggesting that people who tend to experience different patterns of mixed emotions are likely to be more or less sensitive to signals from the body, we cannot exclude this possibility. Additional research is needed to investigate this possibility.

Altogether, the results suggest that although all participants showed a decrease in psychopathological symptoms and an increase in positivity ratio and somatic symptoms between the two time points, the freshmen who declared that experiencing the pattern of secondary mixed emotion was their natural response to loss proved to be more resilient to the stress of the transition than those who experienced positive and negative emotions sequentially. After 6 months of living in a new academic environment, participants with the tendency to experience secondary mixed emotions demonstrated decreased levels of depressive and anxiety symptoms, an increased positivity ratio, and stable somatic health, whereas in participants experiencing opposing emotions sequentially, high levels of depressive and anxiety symptoms, along with a low positivity ratio, remained stable over time, and their somatic complaints increased. However, the conclusion needs to be approached cautiously since one of the limitations of this study involves the size of the compared samples. The lack of significant results for the sequential group might be partially due to the unequal size of the compared group as well as to the small size of the sequential group. Nevertheless, the results seem to provide preliminary empirical evidence that experiencing positive and negative emotions combined into one feeling of a distinctive quality may be a more efficient emotional redemption strategy than providing a positive context for negative affect by experiencing co-occurring opposing emotions.

## General Discussion

It is impossible to avoid worry and anxiety under the pressure of adversity, but a bit of positive affect may help to ameliorate them. Such a mixed emotional response is quite natural because many stressful events consist of not only disadvantages but also opportunities, associations with better circumstances, or a few pleasant or funny elements at the least. Thus, the ability to react with mixed feelings to difficult life episodes might be an efficacious way of regulating distress and, thus, fostering resilience. We proposed that opposite affects can co-occur in the form of separate emotions arising sequentially or simultaneously or can be blended into a single emotion – a secondary mixed emotion. The present studies investigated which types of positive affect and emotional reactions to distress are more adaptive. The three presented studies provided clear and consequent evidence that mixed secondary emotions, represented in our studies by nostalgic experiences, promote coping processes by providing the participants with a higher capacity to handle adversity, as compared with sequentially and simultaneously mixed emotions. In terms of motivational function, which is traditionally seen as predominantly important regarding emotions, secondary mixed emotions produce solution-oriented action rather than avoidance, as well as faster coping resolution. Regarding the informative function, secondary mixed emotions influence thought in a way that is favorable to understanding one’s own experiences. It provides better narrative organization and easier access to self-knowledge. Furthermore, the longitudinal study provided preliminary evidence that individuals characterized by proneness to experiencing secondary mixed emotions are more resilient to the stress of transitions to new life circumstances than those characterized by proneness to feeling positive and negative emotions separately.

The results are congruent with the data concerning a large range of benefits from the example of secondary mixed emotions that was used in our studies – nostalgia. The previous research indicates that the beneficial effects of nostalgia (a reduction of existential anxiety, generation of positive affect, decreased loneliness, and increased self-regard and sense of identity) can be assigned to the content of that emotion rather than to its structure. Namely, the existing data suggest that these effects are caused by the accessibility of representations of positive social connections in the past [68] or by activating meaning-providing structures that serve an existential function [69]. However, the differences between the effects of this secondary mixed emotion and other patterns of mixed emotions, as revealed in our studies, cannot be simply assigned to the content of the emotions, because all of the evoked emotional responses consisted of a sorrowful longing for a beloved person, combined with an affectionate reminiscence of time spent together in the past, and because all of the compared emotional experiences differed only in the way in which the positive and negative affects were combined. Thus, in the presented studies, the advantageous impact on coping and resilience seems to result from the pattern of a mixed emotional experience rather than from its content. What is more, because nostalgia is defined as a positive emotion with tones of loss and is proven to consist of more positive than negative components [23], the results could be attributed to the desirable outcomes of positive affect. However, all of the materials used for eliciting emotional responses in our studies were counterbalanced with respect to positive and negative expressions, and the reported intensities of the feelings induced were similar in various groups. Taken together, the observed effects seem to rely on the pattern of the mixed emotions, rather than on their affective balance, intensities, or contents.

It is possible that the mechanism that underpins the adaptive superiority of secondary mixed emotions over other patterns of mixed emotions may be the lessening of the salience of affective ambivalence. In the case of two inconsistent emotions experienced at the same time, the affective opposition is completely clear, so the resulting tension is relatively high, but in the case of one discrete emotion embracing both conflicting affects, the affective opposition is somehow covert, and consequently, the tension may be lower. According to the psychodynamic view of emotional development, the fusion of opposite affects into a coherent state of mind is an extensive process involving a subsequent freeing of the mental representation of both affects from their extreme forms [70]. Although this idea has not been empirically supported, there are some data confirming the internal self-regulatory processes within representations of the experience of opposite affects. For example, it was shown that mixed emotions were recalled as being less intense than they were when experienced in the past and that their intensity was increasingly underestimated at the time of recall, which did not occur to the same degree with unipolar negative emotions [71]. Thus, it seems that the functionality of mixed emotions may depend on the level of ambivalence and tension resulting from the conflict between co-occurring opposite affects. However, we did not measure these variables, and the exact mechanism through which secondary mixed emotions are associated with more efficient coping is still unknown. Future work should more directly examine whether ambivalence and subsequent tension are a mediating link between various patterns of mixed emotional experience and the capacity to handle adversities and clarify the specific mechanisms by which secondary mixed emotions can benefit resilience.

Accordingly, an important future direction is to examine the exact role of the ambivalence that is produced when positive and negative affects are experienced at the same time. As noted above, we considered ambivalence and tension to be crucial elements of the mechanism underlying the differences in functionality between the patterns of mixed emotions. The uncomfortable character of the co-occurrence of opposite affects is well-known. However, there are reasons to expect some benefits from ambivalence. The experienced conflict, which is produced by the experience of emotions of opposite valence, may facilitate creativity in the face of adversity because emotional disharmony suggests that a situation is extraordinary and remarkable, which stimulates curiosity and initiative [55].

There are some limitations to the presented studies that should be noted. First, there is a lack of previous evidence for the validity of the measure of the tendency to experience secondary mixed emotions used in Study 3. Nevertheless, the assessment of this proneness was clearly useful, as evidenced by the presented results. Still, the assessment should be considered to be at a preliminary stage of development, and further demonstration of its validity and reliability are desirable. Second, our conclusions in the present studies are limited by the inclusion of only one secondary mixed emotion. Thus, the extent to which the reported results can be generalized to all secondary mixed emotions is currently unknown. We acknowledge that secondary emotions need to be examined separately because they are discrete emotional responses with highly specific contents. For this reason, our results concern only one example of the entire category of secondary mixed emotions. Although we put in much effort to compare the emotional responses specifically with respect to their patterns, the question of whether every secondary mixed emotion has the same motivational and informational effects as nostalgia does remains. Hence, further research should include other types of secondary mixed emotions (e.g., poignancy, empathy, awe, or tenderness) and determine whether or not the observed beneficial consequences for coping result from nostalgia *per se* or from the fact that nostalgia is a type of secondary mixed emotion.

Third, there are some potentially important variables that we did not include in our research. For instance, in Study 1 and Study 2, the role of the trait measures of dependent variables was not examined, which presents the possibility that failures in randomization affected the results. The possibility could not be excluded, for instance, that the obtained accessibility to solution-oriented actions is partly affected by the coping style, which was not controlled in our studies. In addition, in Study 3, the trait component of the STAI was not analyzed, but it may be an important co-variant or an additional outcome for ANOVAs. Nevertheless, we were only interested in state functioning, and the trait measure would not be expected to show significant differences over a six-month period. Furthermore, the trait anxiety measure is so closely related to the state anxiety measure that it would be redundant and cause difficulties if used as a co-variant. Finally, in Study 1 and Study 2 despite the fact that the feelings reported by participants while reading the story and their intensities were measured, we also should have assessed other overall valuations of experienced emotional states (i.e., reported by participants affective balance or content of felt emotions). Although including a large number of measurements in one experiment is methodologically difficult, it would help to ensure that evoked in our study three patterns of mixed emotions were similar experiences and differed only in the way in which the opposing affects were blended. Our research is an initial exploration of different patterns of mixed emotions regarding their adaptive consequences, and further studies are required to examine their effects on coping more thoroughly.

The results obtained from our studies provided preliminary empirical evidence that secondary mixed emotions may promote more efficient coping than sequentially and simultaneously mixed emotions. The future direction of research should explore the exact character of this beneficial impact further. We initially proposed that the adaptive consequences of experiencing secondary mixed emotions are related to emotion regulation processes; however, due to the preliminary nature of the presented studies, we did not assess emotion regulation strategies. Thus, in order to draw such conclusions, future research regarding the control of emotion regulation is needed. Moreover, it should be noted that people tend to respond to difficult life situations with a wide variety of emotional experiences. The subject of our investigation was the functionality of the different ways of combining positive and negative effects, and we decided not to examine other possible emotional responses to stress. It would be valuable to investigate the consequences of feeling a single unmixed negative emotion in an adverse situation and to compare it with experiencing different patterns of mixed emotions, which would help to determine whether or not the benefits observed from experiencing secondary mixed emotions can be attributed entirely to the structure of emotional experience and help to understand the role of mixed emotions in the broader context of responding to adversity.

One additional avenue for future research may be the examination of individual differences in the functionality of various patterns of mixed emotions. For instance, recent studies show that low identity continuity [72] and high attachment-related avoidance [68] reduce the benefits of nostalgic experience. It is possible that having low personal resources in terms of connecting the present day with positive memories makes experiencing our example of secondary mixed emotions too ambivalent or not positive enough to foster motivational and cognitive processes. In that case, a less advanced mixed emotional response in the form of separate sadness and love/joy may be more advantageous because it allows an individual to separate good thoughts from bad. Different secondary emotions, such as tenderness or gratitude, may have similar or specific limitations depending on their contents and origins.

In addition, the elicited patterns of mixed emotions were assessed in the manipulation checks via short self-reports only. Although self-reports provide important information about emotional experience, future studies might use a multidimensional approach and additionally use, for example, standardized analyses of facial responses. Further research is required in order to include this indicator of various patterns of mixed emotions. It would be also valuable to examine whether there are specific correlates in the brain for experiencing various patterns of mixed emotional experiences – this could be realized using electroencephalography (EEG) or functional magnetic resonance imaging (fMRI).

Finally, it should be noted that the proneness to feeling secondary mixed emotions might served as a mediator of the relationship between the adjustment to life adversities and other dispositional variables that have important consequences for coping, such as personality traits, attachment patterns, or general self-esteem. Although the results of both presented experiments provided considerable evidence that secondary mixed emotions provide a higher capacity to handle adversity, other important individual dispositions should be measured in order to ascertain the salubrious effects of the ability to feel secondary mixed emotions without any doubt. It would also help to rule out the possibility that the beneficial impact of the proneness in experiencing secondary mixed emotions when adjusting to life’s adversities is merely a consequence or a form of having specific positive personal traits and dispositions. However, because it is not possible to measure all the potentially relevant variables in one study and our main intent was to investigate the dispositional level of feeling mixed emotions diversified in terms of their affective contents, which requires extensive assessment procedures, in the present study, we decided not to examine other potentially relevant variables. Moreover, it should be noted that our work is a preliminary investigation of various mixed emotions patterns regarding coping, and its target was not to thoroughly investigate all of the important relationships involved in this novel and nearly unexplored research field. In order to state more certainly that the proneness of experiencing secondary emotions promotes coping with stress caused by transitions, it would be valuable to conduct a longitudinal study in which one group of freshmen would be trained to respond to negative events by experiencing sequentially mixed emotions and another group to respond with secondary mixed emotions, for example. In addition, future studies are needed to determine the role of secondary mixed emotions and other patterns of mixed emotions as mediating variables in the process of coping.

In conclusion, the results obtained from three presented studies offer preliminary support for the idea that the pattern of secondary mixed emotions may provide individuals with a higher capacity to handle adversity than the other two patterns of mixed emotional experience. In particular, the results indicated that experiencing secondary mixed emotions predominates over sequentially and simultaneously mixed emotions in promoting coping processes through fostering the motivational and informative functions of emotions and that individuals prone to feeling secondary mixed emotions were more resilient to stress caused by transitions than those prone to feeling opposing emotions separately.

## Supporting Information

Supplementary Material S1 **Pattern of mixed emotion manipulation.**
(PPTX)
